# Association of Chinese Visceral Adiposity Index and Carotid Atherosclerosis in Steelworkers: A Cross-Sectional Study

**DOI:** 10.3390/nu15041023

**Published:** 2023-02-18

**Authors:** Xuelin Wang, Zhikang Si, Hui Wang, Rui Meng, Haipeng Lu, Zekun Zhao, Jiaqi Hu, Huan Wang, Jiaqi Chen, Yizhan Zheng, Ziwei Zheng, Yuanyu Chen, Yongzhong Yang, Xiaoming Li, Ling Xue, Jian Sun, Jianhui Wu

**Affiliations:** 1 Department of Epidemiology and Health Statistics, School of Public Health, North China University of Science and Technology, No. 21 Bohai Avenue, Caofeidian New Town, Tangshan 063210, China; 2 Key Laboratory of Coal Mine Health and Safety of Hebei Province, School of Public Health, North China University of Science and Technology, No. 21 Bohai Avenue, Caofeidian New Town, Tangshan 063210, China

**Keywords:** cross-sectional study, CVAI, carotid atherosclerosis, steelworkers

## Abstract

The Chinese Visceral Adiposity Index (CVAI) is an indicator of visceral adiposity dysfunction used to evaluate the metabolic health of the Chinese population. Steelworkers are more likely to be obese due to their exposure to special occupational factors, and have a higher prevalence of carotid atherosclerosis (CAS). This study aimed to analyze the special relationship between CVAI and CAS among steelworkers. A total of 4075 subjects from a northern steel company were involved in the cross-sectional study. Four logistic regression models were developed to analyze the correlation between CVAI and CAS. In addition, the restricted cubic spline was applied to fit the dose–response association between CVAI and CAS risk. In the study, the prevalence of CAS was approximately 25.94%. After adjustment for potential confounders, we observed a positive correlation between CVAI and CAS risk. Compared to the first CVAI quartile, the effect value odds ratio (OR) and 95% CI in the second, third, and fourth CVAI quartile were 1.523 (1.159–2.000), 2.708 (2.076–3.533), and 4.101 (3.131–5.372), respectively. Additionally, this positive correlation was stable in all subgroups except for female. Furthermore, we also found a non-linear relationship between CVAI and CAS risk (*p* nonlinear < 0.05). Notably, CVAI could increase the risk of CAS when higher than 106. In conclusion, our study showed that CVAI might be a reliable indicator to identify high-risk populations of CAS among steelworkers.

## 1. Introduction

With the economic development and aging of the population, the prevalence of cardiovascular disease is increasing. Because of its long course, complex etiology, and many complications, it has become the most important disease affecting the quality of life [1,2]. According to the Global Disease, Injury, and Risk Factor burden study [3], an estimated 422.7 million people worldwide are affected by cardiovascular disease, resulting in about 17.9 million deaths, accounting for 31 percent of global deaths. This causes a huge burden on patients, families, and the national health care system. Atherosclerosis (AS) is a vascular inflammatory disease involving the large and middle arteries and characterized by arterial wall thickening and hardening, and decreased elasticity [4]. Carotid atherosclerosis (CAS) is the manifestation of vascular lesions in the carotid artery and reflects the early stage of AS. CAS has always been considered a risk factor for cardiovascular disease. It can induce malignant cardiovascular events such as myocardial infarction and stroke through vascular wall lesions, changes in blood composition, and hemodynamic changes [5,6,7]. Therefore, the early prevention of CAS is significant for reducing the burden of cardiovascular disease.

Obesity is closely related to CAS; particularly, the correlation between visceral obesity and CAS has aroused widespread attention. Recent studies have shown that visceral adipose tissue (VAT) plays a more important role in the development of metabolic diseases than subcutaneous adipose tissue (SAT) [8,9]. VAT may be an independent risk factor for the atherosclerosis risk [10]. Magnetic resonance imaging (MRI) and computed tomography (CT) are commonly used to measure visceral fat [9]. However, these are used for special clinical purposes, and have the disadvantages of being time-consuming and expensive, and exposing radiation to subjects and operators. In addition, existing obesity indicators such as body mass index (BMI) and waist circumference (WC) do not distinguish subcutaneous adipose and visceral adipose. Therefore, many indicators which can effectively assess visceral obesity have been continuously developed. The Chinese Visceral Adiposity Index (CVAI) is an index used to estimate visceral obesity based on the body fat characteristics of the Asian population [11]. It combines WC, BMI, Triglyceride (TG), and high-density lipoprotein cholesterol (HDL-C), and takes into account the effects of gender and age. Notably, as a new indicator of visceral obesity, CVAI has been proven to be an independent predictive indicator of diabetes and cardiovascular diseases [12,13,14,15]. Therefore, it is necessary to explore the relationship between CVAI and CAS in order to better achieve the early prevention of CAS.

As steel industry in an important pillar industry in China, the health of steelworkers cannot be ignored. According to related studies, steelworkers may have a higher prevalence of CAS compared with the general population due to their exposure to special risk factors such as high temperature, noise, and shift work in the production environment [16,17,18,19]. Therefore, it is particularly important to find a simple and effective predictor. However, there are no studies on the relationship between CVAI and CAS in steelworkers. The purpose of this study is to explore the relationship between CVAI and CAS in steelworkers.

## 2. Materials and Methods

### 2.1. Study Design and Participants

**Figure 1:**
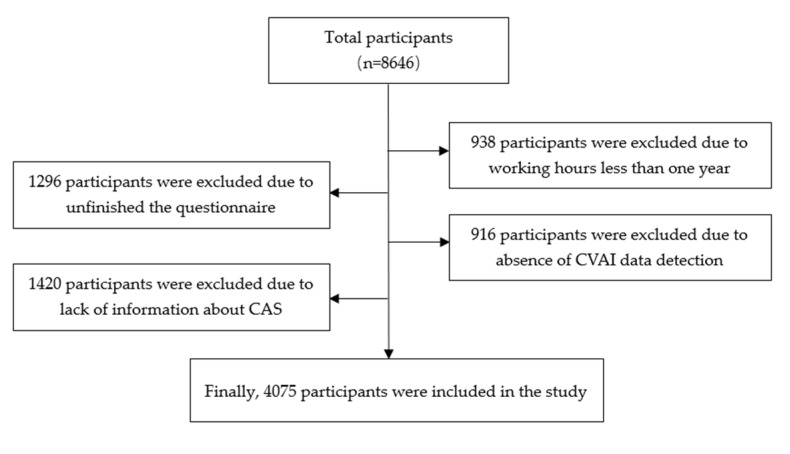
The process of study participants selection. CVAI: Chinese Visceral Adiposity Index, CAS: carotid atherosclerosis.

This is a large cross-sectional study. A total of 4075 workers from a steel company who attend occupational examination and physical examination between March and June 2017 were selected as study participants. The process of study participants selection is shown in Figure 1. Participants who were under the age of 18 and had less than one year of service were excluded. Participants who missed the information about CAS and had incomplete CVAI data detection were excluded. All participants signed the informed consent for inclusion before they participated in the study.

### 2.2. Date Collection

There were three types of information collected in this study, which were questionnaire information, physical examination information, and laboratory examination information. All information was obtained by specially trained researchers following the uniform working standard.

The questionnaire included general demographic characteristics (age, gender, marital status, education status, and income status), history of personal and family disease (hypertension, diabetes, dyslipidemia, and atherosclerosis), behavioral lifestyle (smoking, drinking, diet, and physical exercise), and work conditions (length of service, shifts, exposure to high temperature, noise, and other harmful factors). The physical examination included the height, weight, blood pressure, and waist circumference of participants.

We took study subjects’ venous blood samples in the early morning after fasting for 12 h, and tested the blood biochemical indicators such as fasting glucose, TG, HDL-C, low-density lipoprotein cholesterol (LDL-C), total cholesterol (TC), homocysteine, and uric acid (UA) using the Myriad Automatic Biochemistry Analyzer (BS-800).

### 2.3. Assessment of CAS

The study participants were examined for CAS using a portable color Doppler ultrasound system with a probe frequency of 5 to 12 MHz to measure intima-media thickness (IMT). The measurements were performed with the participants in a supine position, starting from the heel of the neck and proceeding upward to the common carotid artery (CCA), internal carotid artery (ICA), external carotid artery (ECA), and bifurcation. According to the 2017 Chinese Consensus on the Diagnosis of Head and Neck Atherosclerosis [20], the diagnostic criteria for CAS were a carotid IMT ≥ 1.0 mm on the color Doppler ultrasound.

### 2.4. Assessment of CVAI

Male: CVAI= −267.93 + 0.68 × age (years) + 0.03 × BMI (kg/m^2^) + 4.00 × WC (cm) + 22.00 × Log10 TG (mmol/L) −16.32 × HDL-C (mmol/L);

Female: CVAI= −187.32 + 1.71 × age (years) + 4.23 × BMI (kg/m^2^) + 1.12 × WC (cm) + 39.76 × Log10 TG (mmol/L) −11.66 × HDL-C (mmol/L).

### 2.5. Assessment of Covariates

Hypertension: according to the Chinese Guidelines for the Prevention and Treatment of Hypertension 2018 Revised Edition [21], the diagnostic criteria for hypertension in this study were systolic blood pressure ≥ 140 mmHg and/or diastolic blood pressure ≥ 90 mmHg, or a previous history of hypertension and currently taking antihypertensive medication.

Dyslipidemia: dyslipidemia usually refers to elevated or reduced serum levels of cholesterol and/or triglycerides. According to the Guidelines for the Prevention and Treatment of Dyslipidemia in Chinese Adults (2016 Revised Edition) [22], the diagnostic criteria for dyslipidemia in this study were serum TC concentration ≥6.22 mmol/L, and/or LDL-C concentration ≥4.14 mmol/L, and/or TG concentration ≥2.26 mmol/L, and/or HDL-C concentration <1.04 mmol/L, or previous history of hyperlipidemia and currently taking lipid-lowering drugs.

Diabetes mellitus: According to the Chinese Guidelines for the Prevention and Treatment of Type 2 Diabetes Mellitus (2020 Edition) [23], the diagnostic criteria for diabetic disease in this study were fasting blood glucose (FPG) ≥ 7.0 mmol/L or a previous history of diabetes and currently receiving treatment.

Physical exercise: The physical activity of the study participants was investigated and classified as mild, moderate, or severe based on the International Physical Activity Scale (long-roll version) [24].

Diet: The frequency of consumption of eight food groups were investigated in the study, including whole grains, vegetables, fruits, low-fat milk, nuts and legumes, sweet drinks, red meat and processed meat products, and sodium intake. Dietary scores were calculated according to Dietary Approaches to Stop Hypertension (DASH). In this study, dietary profiles were classified into DASH scores < 25 and ≥25 according to the median of the DASH dietary pattern score.

Smoking: Smoking was defined as smoking at least one cigarette per day for more than six months in this study. Smoking status was categorized as never smoked, former smokers, and current smokers.

Drinking: Drinking was defined as drinking alcohol at least once a week for more than six months in this study. Drinking status was categorized as never drinking, former drinkers, and current drinkers.

Occupational factors: Defined according to the contents of occupational factors such as high temperature, noise, and shift work in the questionnaire for steelworkers.

### 2.6. Statistical Analysis

SPSS 23.0 and R 4.1.2 were used for statistical analysis in the study. *p* < 0.05 (bilateral) was considered statistically significant. The general characteristics of the study subjects were described based on the quartiles of CVAI, and normality tests were performed for all measurement data. The measurement data obeying the normal distribution were expressed as $\bar{x}\pm s$, and the ANOVA were used for comparison between groups. The non-normally distributed measurement data were represented by M (P25, P75), and the Kruskal–Wallis H test was used for comparison between groups. The count data were used as the ratio, and the Pearson χ2 test was used for comparison between groups. 

Considering the relationship between CVAI and CAS might be confounded in univariate analysis. In this study, we firstly screened possible confounders and then used multifactorial logistic regression to assess the relationship between CVAI and CAS. Four models were developed; model 1 was a crude model. Model 2 adjusted for age, sex, education, marital status, income status, smoking, drinking, physical exercise, and diet. Model 3 further adjusted for hypertension, diabetes, hyperlipidemia, uric acid, homocysteine, hypersensitive C-reactive protein (hs-CRP), and white blood cell (WBC). Model 4 continued to adjust for exposure to high temperature, noise, and shifts. The variables included in models all satisfied the tolerance > 0.1 and the variance inflation factor (VIF) < 10. The linear relationship between the continuous independent variables and the logit-transformed values of the dependent variable were tested by the Box–Tidwell test in logistic regression models. We also estimated the relationship between each 1-SD increase in CVAI and CAS prevalence. The dose–response relationship between CVAI and CAS risk was fitted using a restricted cubic spline model incorporating logistic regression (the 25th, 50th, and 75th percentiles were selected as the nodes). In addition, we also evaluated the incremental predictive value of CVAI in the CAS risk assessment among steelworkers. To further validate the robustness of the results, we performed subgroup analyses, including age (<50 vs. ≥50), sex (male vs. female), hypertension (Yes vs. No), diabetes (Yes vs. No), dyslipidemia (Yes vs. No), high temperature exposure (Yes vs. No), noise exposure (Yes vs. No), shift status (Yes vs. No), smoking (Yes vs. No), and drinking (Yes vs. No).

## 3. Results

### 3.1. General Characteristics of the Study Subjects 

The study ultimately included 4075 participants, 3720 men and 355 women, with a mean age of 49.2 ± 7.9 years. In the 4075 study participants, 1057 had carotid atherosclerosis, with a CAS prevalence of 25.94%. The prevalence of CAS in the first, second, third, and fourth CVAI quartile were 10.9%, 18.5%, 30.5%, and 43.8% respectively. The general characteristics of participants based on CVAI quartiles are shown in Table 1. The participants in different CVAI quartiles differed in age, gender, family history of CAS, education status, marital status, smoking, drinking, diet, shifts status, hypertension, diabetes, dyslipidemia, BMI, WC, TG, FPG, SBP, DBP, UA, Hcy, hs-CRP, and WBC. And the differences were statistically significant (*p* < 0.05). However, there were no significant differences in income status, physical exercise, high temperature exposure, noise exposure, LDL-C, and TC.

### 3.2. Analysis of Risk Factors for CAS among Steelworkers

The analysis of possible risk factors for CAS among steelworkers is shown in Table 2. We found that age, BMI, WC, UA, Hcy, and WBC were positively correlated with CAS prevalence, and the effect values of OR (95% CI) were 1.105 (1.092–1.117), 1.104 (1.082–1.126), 1.063 (1.054–1.071), 1.001 (1.000–1.002), 1.008 (1.003–1.008), and 1.125 (1.018–1.171), respectively. Men had a higher risk compared to women, with an OR (95% CI) of 3.147 (2.231–4.440). Participants with a family history of CAS had a higher risk compared to those without, with an OR (95% CI) of 3.555 (2.311–5.467). Participants with medium and high education had a lower risk compared to those with low education, with an OR (95%CI) of 0.768 (0.651–0.906) and 0.289 (0.228–0.365). The married participants seemed to have a higher risk compared with unmarried, with an OR (95% CI) of 2.185 (1.369–3.485). Among different incomes, the participants with a per capita monthly household income <1000 had a higher risk compared with those with an income ≥3000. Participants who smoked and drank had a higher risk of CAS, with an OR (95% CI) of 1.698 (1.461–1.975) and 1.555 (1.345–1.797). Participants with heavy physical exercise had a lower risk of CAS compared with mild. Participants with a DASH score ≥25 have a lower risk of CAS compared with <25, with an OR (95% CI) of 0.827 (0.719–0.952). Additionally, participants with exposure to noise, high temperature, and shifts had a higher risk of CAS, with an OR (95% CI) of 1.162 (1.01–1.337), 1.522 (1.322–1.753), and 1.311 (1.079–1.594). In addition, we also found participants with hypertension, diabetes, and dyslipidemia had a higher risk of CAS, and their effect values of OR (95%CI) were 3.244 (2.8–3.758), 1.95 (1.604–2.369), and 1.8 (1.56–2.076), respectively.

### 3.3. The Relationship between CVAI and CAS among Steelworkers 

**Figure 2:**
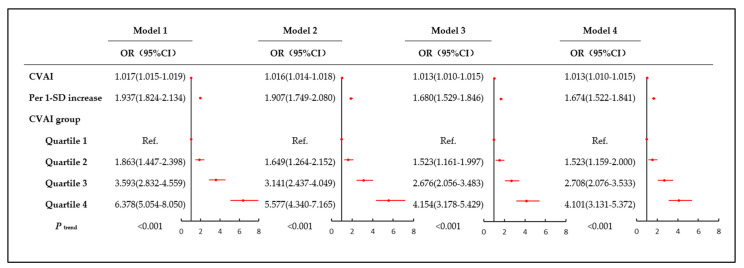
Association between CVAI and the risk of CAS prevalence. CVAI: Chinese Visceral Adiposity Index, CAS: carotid atherosclerosis, OR: odds ratio, CI: confidence interval. Model 1: crude model; Model 2: adjusted for age, sex, family history of CAS, education, marital status, income status, smoking, drinking, physical exercise, and diet; Model 3: further adjusted for hypertension, diabetes, dyslipidemia, uric acid, homocysteine, WBC, and hs-CRP; Model 4: further adjusted for exposure to high temperature, noise, and shifts. The linear trends between CVAI quartiles were assessed by entering the median of each quartile.

We developed four logistic regression models to analyze the relationship between CVAI and CAS (Figure 2). The effect value of the model can be interpreted as: with the increase in CVAI, the probability of CAS prevalence increases correspondingly. When CVAI was a continuous variable, CVAI increased by one unit, with the corresponding effect value OR (95% CI) in the four models being 1.017 (1.015–1.019), 1.016 (1.014–1.018), 1.013 (1.010–1.015), and 1.013 (1.010–1.015), respectively. When CVAI was the grouping variable, the risk of CAS prevalence increased with increasing CVAI levels in the four models (*p* trend values < 0.001). In model 1, the OR (95% CI) of CAS prevalence in the second, third, and fourth CVAI quartile were 1.863 (1.447–2.398), 3.593 (2.832–4.559), and 6.378 (5.054–8.050), respectively. In model 2, the OR (95% CI) of CAS prevalence in the second, third, and fourth CVAI quartile were 1.649 (1.264–2.152), 3.141 (2.437–4.049), and 5.577 (4.340–7.165), respectively. In model 3, the OR (95% CI) of CAS prevalence in the second, third, and fourth CVAI quartile were 1.523 (1.161–1.997), 2.676 (2.056–3.483), 4.154 (3.178–5.429), respectively. In model 4, the OR (95% CI) of CAS prevalence in the second, third, and fourth CVAI quartile were 1.523 (1.159–2.000), 2.708 (2.076–3.533), and 4.101 (3.131–5.372), respectively. In addition, when CVAI increased by 1-SD, the OR (95% CI) were 1.937 (1.824–2.134), 1.907 (1.749–2.080), 1.680 (1.529–1.846), and 1.674 (1.522–1.841) in the four models, respectively.

**Figure 3:**
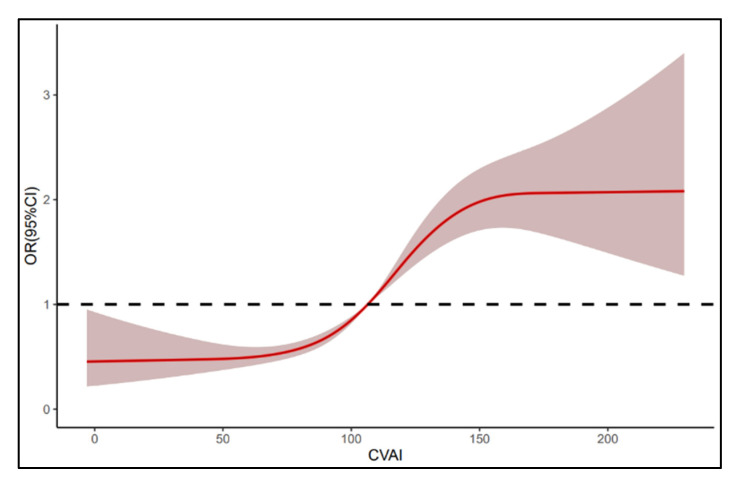
Dose–response relationship of CVAI and CAS risk. CVAI: Chinese Visceral Adiposity Index, CAS: carotid atherosclerosis, OR: odds ratio, CI: confidence interval.

To describe the relationship between CVAI and CAS more intuitively, we fitted a smooth curve of CVAI and CAS risk using the restricted cubic spline model incorporating logistic regression. After adjusting for age, sex, education, marital status, income status, smoking, drinking, physical exercise, diet, hypertension, diabetes, dyslipidemia, uric acid, homocysteine, WBC and hs-CRP, high temperature, noise, and shifts, we found a nonlinear positive relationship between CVAI and CAS risk (*p* nonlinear < 0.05). We also found that CVAI could increase CAS risk when higher than 106 (Figure 3).

### 3.4. Subgroup Analysis

In the subgroup analysis, we used model 4 to analyze the relationship between CVAI and CAS and adjusted all the confounders of model 4 except for the stratified factors. The results are shown in Table 3; we found that the positive correlations between CVAI and risk of CAS prevalence among steelworkers were consistent across the subgroups, including age (<50 vs. ≥50), hypertension (Yes vs. No), diabetes (Yes vs. No), dyslipidemia (Yes vs. No), high temperature exposure (Yes vs. No), noise exposure (Yes vs. No), shift status (Yes vs. No), smoking (Yes vs. No), and drinking (Yes vs. No). Unfortunately, there were no positive correlations between CVAI and risk of CAS prevalence among women.

### 3.5. Incremental Predictive Value of CVAI in Risk Assessment of CAS among Steelworkers

The incremental predictive values of CVAI in the risk assessment of CAS are summarized in Table 4. The C-statistics of the basic model were significantly enhanced by adding CVAI (*p* < 0.001). In addition, after adding CVAI to the basic model, the integrated discrimination improvement (IDI) showed a significant increase of 3.13% (2.51–3.75%) and the net classification index (NRI) showed a significant increase of 7.14% (3.66–10.62 %). 

## 4. Discussion

This study included 4075 study subjects with a CAS prevalence of 25.94%. We found that the risk of CAS prevalence increased with increasing CVAI. Additionally, there was a nonlinear positive relationship between CVAI and CAS, with a much higher risk of CAS when CVAI was higher than 106. After being stratified by age (<50 vs. ≥50), sex (male vs. female), hypertension (Yes vs. No), diabetes (Yes vs. No), dyslipidemia (Yes vs. No), high temperature exposure (Yes vs. No), noise exposure (Yes vs. No), shift status (Yes vs. No), smoking (Yes vs. No), and drinking (Yes vs. No), this trend persisted in all subgroups except for women. Our findings suggest that CVAI may be a reliable predictor of carotid atherosclerosis among steelworkers.

Obesity is a major public health problem worldwide [25]. According to the 2020 report on the nutrition and chronic disease status of the Chinese population [26], the current prevalence of overweight and obesity among Chinese adults has reached 50.7%. More and more studies suggest that VAT is a key predictor of health risks associated with obesity, and it plays a critical role in cardiometabolic disease [27,28,29,30,31]. Visceral obesity is the result of a pathological response of abdominal adipose tissue to caloric homeostasis in susceptible individuals and contributes directly or indirectly to the development of metabolic cardiovascular disease [27]. Because of the complexity of VAT imaging measurement, some indices which can effectively assess the area of VAT have been continuously developed. Although BMI and WC are recommended by WHO for obesity, they do not take into account the heterogeneity of adipose deposition. The increase in BMI and WC may be due to the deposition of subcutaneous adipose tissue. CVAI is an index for estimating visceral obesity based on Asian people, and external verification shows that it is positively correlated with VAT. Current studies show that CVAI performs well as an index of visceral obesity in predicting cardiovascular disease in the general population, and is superior to BMI, WC, and VAI [32,33,34]. However, the relationship between CVAI and CAS is unknown in steelworkers. Considering that steelworkers, as an occupational group, are more likely to be overweight and obese compared with the general population due to their specific occupational factors [35,36,37], studies on the general population may not be applicable to steelworkers. Our study adds to the evidence that CVAI is associated with CAS in steelworkers and remains stable in subgroup analysis.

Several possible mechanisms have been proposed to explain how VAT affects the occurrence and development of atherosclerosis. First, since most VAT is drained from the portal vein, adipocytes in VAT can secrete large amounts of lipids, exposing the liver to high concentrations of free fatty acids and triglycerides, leading to hepatic metabolic damage, further triggering disorders of glucolipid metabolism and promoting the development of atherosclerosis [38]. Second, excessive VAT accumulation triggers inflammation, as hypertrophic adipocytes contain a large number of macrophages, which secrete large amounts of inflammatory cytokines such as tumor necrosis factor alpha and interleukin 6, and inhibit the secretion of lipocalin [39,40,41]. Lipocalin is an anti-atherosclerotic, antidiabetic and anti-inflammatory substance. Inflammation is an important factor in the development of atherosclerosis. Third, VAT is a marker of increased ectopic fat. Ectopic fat deposition is defined as the accumulation of excess adipose tissue around normal lean tissue in the liver, heart (pericardium, epicardium, and within the myocardium), and skeletal muscle. Epicardial and perivascular adipose tissue exerts direct pathogenic effects on the myocardium, coronary arteries, and peripheral vasculature by secreting local vasoactive and inflammatory factors, which may lead to atherosclerosis [42,43].

Our study shows that there is a non-linear positive dose–response relationship between CVAI and CAS, and when CVAI is higher than 106, the risk of CAS is greatly increased. Several studies have examined the association of CVAI with CAS. A retrospective study [44] shows that of the 4437 middle-aged and elderly participants enrolled, 2162 had CAS. After adjusting for age, sex, hypertension, and T2DM, CVAI was independently associated with CAS (OR 1.39, 95% CI 1.21, 1.61). The cut-off value to distinguish between CAS and non-CAS was 96.51. A study in Taiwan [44] showed that CVAI, a novel indicator of abdominal obesity, performed better compared with traditional indicators such as BMI and WC in assessing the risk of atherogenic cardiovascular disease, especially among women. The optimal CVAI cutoff value for men and women were 83.7 and 70.8, respectively. Hu [45] also observed a significant association between CVAI and carotid plaque risk. Each increase in 1-SD in CVAI increased the risk of carotid plaque by 53% (HR 1.53, 95%CI 1.48–1.59). The appropriate cut-off value of CVAI in the population was 86.229. These studies and our results all suggest that CVAI may be a reliable indicator of carotid atherosclerosis. However, the difference is that in our study, the cut-off value of CVAI was higher, which may be due to the fact that our study subjects were steelworkers. It has been shown that steelworkers have a higher prevalence of overweight and obesity than the general population due to their special occupational factors such as shift work, noise, and occupational stress in their production and living environment [46,47,48]. In addition, most of the steelworkers are middle-aged men. Therefore, the CVAI of steelworkers is overall higher than that of the general population.

The main strength of our study was the sufficiently large sample size; we ultimately included 4075 study subjects. The relationship between CVAI and CAS remained after adjusting many confounding factors. In addition to traditional risk factors of CAS, we also considered the impact of occupational exposure, such as shift work. A study reported that shift workers’ behavioral and environmental cycles are typically misaligned relative to their endogenous circadian system, predisposing an individual for poor metabolic health [49]. Furthermore, we conducted a subgroup analysis of the sensitive population. Therefore, the findings of our study can be considered relatively reliable. However, our study also has some limitations. First, because our study was a cross-sectional study, causal inference could not be made. The causal relationship between CVAI and CAS needs to be further confirmed in a prospective cohort study. Second, because our study population was employed steelworkers, it is also unknown whether our study results can be generalized to other populations. In addition, due to the small number of women in our study population, it may not provide sufficient statistical power to adequately examine the relationship between CVAI and CAS among women. The presence of genetic alterations in insulin signaling can increase the risk of cardio-vascular events through endothelial insulin resistance and inflammation [50], but we have not considered it in our study. Because of these limitations, our study only provides a hypothesis; the role of CVAI in the process of carotid atherosclerosis needs to be further investigated and confirmed in more studies.

## 5. Conclusions

CVAI might be a reliable indicator to identify individuals at high risk for CAS among steelworkers. Individuals with CVAI higher than 106 should receive additional screening and preventive intervention for CAS.

## Figures and Tables

**Table 1:** General characteristics of the study subjects.

**Variables**	CVAI				*p* Value
	Quartile 1 (*n* = 1019)	Quartile 2 (*n* = 1019)	Quartile 3 (*n* = 1019)	Quartile 4 (*n* = 1018)	
**CVAI range**	<78.36	78.36~106.42	106.42~134.01	>134.01	
**Age (years)** ^**a**^	47.9 ± 8.2	49.1 ± 8.0	49.7 ± 7.7	50.2 ± 7.8	<0.001
**Gender: Male (%)**	807 (79.2)	934 (91.7)	973 (95.5)	1006 (98.8)	<0.001
**Family history of** **CAS: yes (%)**	16 (1.6)	25 (2.5)	28 (2.7)	37 (3.6)	<0.001
**Education status (%)**					0.004
Low	199 (19.5)	221 (22.7)	243 (23.8)	262 (25.7)	
Medium	559 (54.9)	567 (55.6)	530 (52.0)	553 (54.3)	
High	261 (25.6)	231 (22.7)	246 (24.2)	203 (20.0)	
**Marital status (%)**					<0.001
Unmarried	60 (5.9)	37 (3.6)	25 (2.5)	27 (2.7)	
Married	923 (90.6)	955 (93.7)	969 (95.1)	962 (94.5)	
Other	36 (3.5)	27 (2.6)	25 (2.5)	29 (2.8)	
**Income status (%)**					0.256
<1000 yuan	91 (8.9)	102 (10.0)	72 (7.1)	98 (9.8)	
1000 ~3000 yuan	817 (80.2)	811 (79.6)	847 (83.1)	822 (80.7)	
≥3000 yuan	111 (10.9)	106 (10.4)	100 (9.8)	98 (9.6)	
**Smoking status (%)**					<0.001
Never	544 (53.4)	475 (46.6)	378 (37.1)	353 (34.7)	
Once	39 (3.8)	58 (5.7)	73 (7.2)	76 (7.5)	
Now	436 (42.8)	486 (47.7)	568 (55.7)	589 (57.9)	
**Drinking status (%)**					<0.001
Never	687 (67.4)	605 (59.4)	553 (54.3)	551 (54.1)	
Once	17 (1.7)	25 (2.5)	32 (3.1)	40 (3.9)	
Now	315 (30.9)	389 (38.2)	434 (42.6)	427 (41.9)	
**Physical exercise (%)**					0.065
Mild	38 (3.7)	54 (5.3)	63 (6.2)	56 (5.5)	
Moderate	88 (8.6)	89 (8.7)	99 (9.7)	113 (11.1)	
Severe	893 (87.6)	876 (86.0)	857 (84.1)	849 (83.4)	
**Diet (%)**					0.001
DASH score < 25	265 (26.0)	313 (30.7)	308 (30.2)	348 (34.2)	
DASH score ≥ 25	754 (74.0)	706 (69.3)	711 (69.8)	670 (65.8)	
**High temperature** **Exposure: yes (%)**	415 (40.7)	498 (48.9)	501 (49.2)	572 (56.2)	<0.001
Noise exposure: yes (%)	454 (44.6)	463 (45.4)	471 (46.2)	473 (46.5)	0.821
**Shift status (%)**					0.196
Never	203 (19.9)	181 (17.8)	193 (18.9)	159 (15.6)	
Once	201 (19.7)	191 (18.7)	184 (18.1)	194 (19.1)	
Now	615 (60.4)	647 (63.5)	642 (63.0)	665 (65.3)	
**Hypertension: yes (%)**	161 (15.8)	241 (23.7)	335 (32.9)	495 (48.6)	<0.001
Diabetes: Yes (%)	39 (3.8)	111 (10.9)	163 (16.0)	193 (19.0)	<0.001
**Dyslipidemia: yes (%)**	176 (17.3)	311 (30.5)	428 (42.0)	561 (55.1)	<0.001
**CAS: yes (%)**	111 (10.9)	189 (18.5)	311 (30.5)	446 (43.8)	<0.001
**BMI (kg/m^2^)** ^**a**^	22.8 ± 2.4	24.9 ± 2.3	26.4 ± 2.5	29.1 ± 3.6	<0.010
**WC (cm)** ^**a**^	78.3 ± 5.4	86.6 ± 3.5	92.0 ± 3.3	101.2 ± 5.9	<0.001
**HDL-C (mmol/L)** ^**a**^	1.50 ± 0.4	1.3 ± 0.3	1.2 ± 0.3	1.1 ± 0.2	<0.001
**TG (mmol/L)** ^**b**^	0.88 (0.66–1.21)	1.18 (0.89–1.67)	1.52 (1.08–2.15)	1.85 (1.29–2.75)	<0.001
**LDL-C (mmol/L)** ^**a**^	3.1 ± 0.8	3.3 ± 0.9	3.3 ± 0.9	3.3 ± 0.9	0.179
**TC (mmol/L)** ^**a**^	4.9 ± 0.9	5.1 ± 1.0	5.2 ± 1.0	5.3 ± 1.0	0.423
**FPG (mmol/L)** ^**b**^	5.6 (5.3–5.9)	5.8 (5.4–6.2)	5.9 (5.5–6.4)	6.0 (5.6–6.6)	<0.001
**SBP (mmHg)** ^**a**^	124.62 ± 11.9	127.4 ± 12.5	129.3 ± 13.2	134.7 ± 14.9	<0.001
**DBP (mmHg)** ^**a**^	80.4 ± 7.6	82.4 ± 7.8	83.5 ± 8.4	85.4 ± 9.3	<0.001
**UA (μmol/L)** ^**a**^	346.4 ± 88.7	373.5 ± 87.7	397.1 ± 89.8	416.8 ± 92.2	<0.001
**Hcy (μmol/L)** ^**b**^	11.5 (9.6–15.7)	12.1 (10.2–16.9)	12.4 (10.1–17.0)	12.4 (10.5–17.1)	<0.001
**hs-CRP (mg/L)** ^**b**^	0.00 (0.00–0.02)	0.01 (0.00–0.05)	0.02 (0.00–0.07)	0.04 (0.01–0.13)	<0.001
**WBC (10** ^**9**^ **/L)** ^**a**^	6.3 ± 1.6	6.5 ± 1.6	6.9 ± 1.8	7.1 ± 1.7	<0.001

^b^ Data are median (IQR); ^a^ Data are mean ± standard deviation. CVAI: Chinese Visceral Adiposity Index, CAS: carotid atherosclerosis, BMI: body mass index, WC: waist circumference, SBP: systolic blood pressure, DBP: diastolic blood pressure, TC: total cholesterol, TG: triglyceride, HDL-C: high-density lipoprotein cholesterol, LDL-C: low-density lipoprotein cholesterol, FBG: fasting blood glucose, UA: uric acid, Hcy: homocysteine, hs-CRP: hypersensitive C-reactive protein, WBC: white blood cell.

**Table 2:** Risk factors analysis of CAS among steelworkers.

**Variables**	*β*	*SE*	Wald χ2	OR (95%CI)	*p* Value
**Age**	0.100	0.006	295.063	1.105 (1.092–1.117)	<0.001
**Gender**					
Female				1	
Male	1.147	0.176	42.648	3.147 (2.231–4.440)	<0.001
**Family history of CAS**					
No				1	
Yes	1.268	0.220	33.339	3.555 (2.311–5.467)	<0.001
**Education status**					
Low				1	
Medium	−0.264	0.084	9.858	0.768 (0.651–0.906)	0.002
High	−1.243	0.120	107.946	0.289 (0.228–0.365)	<0.001
**Marital status**					
Unmarried				1	
Married	0.781	0.238	10.754	2.185 (1.369–3.485)	<0.001
Other	0.787	0.315	6.239	2.197 (1.185–4.075)	0.012
**Income status**					
<1000 yuan				1	
1000 ~3000 yuan	−0.210	0.121	3.024	0.810 (0.639–1.027)	0.082
≥3000 yuan	−0.480	0.166	8.379	0.619 (0.447–0.857)	0.004
**Smoking status**					
Never				1	
Once	0.934	0.144	42.022	2.544 (1.918–3.373)	<0.001
Now	0.530	0.077	47.533	1.698 (1.461–1.975)	<0.001
**Drinking status**					
Never				1	
Once	1.125	0.194	33.599	3.018 (2.106–4.508)	<0.001
Now	0.441	0.074	35.758	1.555 (1.345–1.797)	<0.001
**Physical exercise**					
Mild				1	
Moderate	0.050	0.173	0.084	1.051 (0.749–1.476)	0.772
Severe	−0.869	0.145	35.765	0.419 (0.315–0.558)	<0.001
**Diet status**					
DASH score < 25				1	
DASH score ≥ 25	−0.190	0.072	6.956	0.827 (0.719–0.952)	0.008
**Noise exposure**					
No				1	
Yes	0.150	0.072	4.411	1.162 (1.01–1.3370)	0.036
**High temperature** **exposure**					
No	0.420	0.072	34.048	1	
Yes				1.522 (1.322–1.753)	<0.001
**Shift status**					
Never				1	
Once	0.266	0.121	4.846	1.304 (1.030–1.653)	0.028
Now	0.271	0.100	7.403	1.311 (1.079–1.594)	0.007
**Hypertension**					
No				1	
Yes	1.177	0.075	245.829	3.244 (2.8–3.758)	<0.001
**Diabetes**					
No				1	
Yes	0.668	0.099	45.083	1.95 (1.604–2.369)	<0.001
**Dyslipidemia**					
No				1	
Yes	0.588	0.073	65.061	1.800 (1.560–2.076)	<0.001
**CVAI**	0.016	0.001	288.446	1.017 (1.015–1.019)	<0.001
**BMI (kg/m**^**2**^)	0.099	0.010	95.261	1.104 (1.082–1.126)	<0.001
**WC (cm)**	0.061	0.004	222.768	1.063 (1.054–1.071)	<0.001
**UA (μ mol/L)**	0.001	0.001	7.214	1.001 (1.000–1.002)	<0.001
**Hcy (μ mol/L)**	0.008	0.003	9.107	1.008 (1.003–1.008)	0.003
**hs-CRP (mg/L)**	0.132	0.08	2.694	1.141 (0.975–1.335)	0.101
**WBC (10** ^**9**^ **/L)**	0.118	0.020	33.492	1.125 (1.018–1.171)	<0.001

OR: odds ratio, CI: confidence interval, CVAI: Chinese Visceral Adiposity Index, CAS: carotid atherosclerosis, BMI: body mass index, WC: waist circumference, UA: uric acid, Hcy: homocysteine, hs-CRP: hypersensitive C-reactive protein, WBC: white blood cell.

**Table 3:** Subgroup analysis of different CVAI levels and risk of CAS prevalence.

Subgroup	Quartile 1	Quartile 2		Quartile 3		Quartile 4	
	OR	OR (95%CI)	*p* Value	OR (95%CI)	*p* Value	OR (95%CI)	*p* Value
**Age**							
<50	Ref.	1.869 (1.162–3.006)	0.010	2.360 (1.459–3.818)	<0.001	4.072 (2.520–6.580)	<0.001
≥50	Ref.	1.688 (1.213–2.294)	0.002	2.808 (2.057–3.832)	<0.001	4.342 (3.163–5.962)	<0.001
**Sex**							
Male	Ref.	1.615 (1.225–2.129)	0.001	2.508 (1.916–3.283)	<0.001	3.938 (3.005–5.161)	<0.001
Female	Ref.	2.377 (0.897–6.304)	0.082	4.434 (1.487–13.226)	0.008	5.173 (0.908–29.481)	0.064
**Hypertension**							
Yes	Ref.	1.715 (1.042–2.821)	0.034	2.655 (1.648–4.278)	<0.001	3.716 (2.324–5.941)	<0.001
No	Ref.	1.651 (1.204–2.265)	0.002	2.482 (1.809–3.406)	<0.001	4.368 (3.156–6.044)	<0.001
**Diabetes**							
Yes	Ref.	2.344 (0.920–5.974)	0.074	2.779 (1.118–6.911)	0.028	4.356 (1.746–10.867)	<0.001
No	Ref.	1.641 (1.239–2.174)	0.001	2.587 (1.961–3.414)	<0.001	4.183 (3.158–5.541)	<0.001
**Dyslipidemia**							
Yes	Ref.	1.633 (0.972–2.745)	0.064	2.523 (1.544–4.123)	<0.001	3.856 (2.375–6.262)	<0.001
No	Ref.	1.742 (1.275–2.381)	0.001	2.661 (1.939–3.650)	<0.001	4.203 (3.027–5.836)	<0.001
**High temperature** **exposure**							
Yes	Ref.	1.813 (1.235–2.663)	0.002	2.780 (1.896–4.076)	<0.001	4.657 (3.187–6.806)	<0.001
No	Ref.	1.608 (1.112–2.326)	0.040	2.495 (1.744–3.571)	<0.001	3.566 (2.453–5.184)	<0.001
**Noise exposure**							
Yes	Ref.	1.846 (1.263–2.697)	0.002	2.378 (1.631–3.466)	<0.001	3.626 (2.477–5.307)	<0.001
No	Ref.	1.639 (1.128–2.381)	0.010	2.921 (2.029–4.205)	<0.001	4.554 (3.140–6.603)	<0.001
**Shift status**							
Yes	Ref.	1.527 (1.102–2.117)	0.011	2.180 (1.577–3.013)	<0.001	3.673 (2.658–5.076)	<0.001
No	Ref.	2.068 (1.307–3.272)	0.002	3.611 (2.310–5.643)	<0.001	4.974 (3.125–7.916)	<0.001
**Smoking**							
Yes	Ref.	1.868 (1.284–2.719)	0.001	2.805 (1.953–4.031)	<0.001	4.607 (3.194–6.645)	<0.001
No	Ref.	1.527 (1.049–2.222)	0.027	2.479 (1.698–3.619)	<0.001	3.578 (2.432–5.263)	<0.001
**Drinking**							
Yes	Ref.	1.947 (1.258–3.015)	0.003	2.867 (1.873–4.387)	<0.001	5.880 (3.826–9.037)	<0.001
No	Ref.	1.552 (1.109–2.171)	0.010	2.476 (1.776–3.454)	<0.001	3.110 (2.213–4.371)	<0.001

CVAI: Chinese Visceral Adiposity Index, CAS: carotid atherosclerosis, OR: odds ratio, CI: confidence interval. In the subgroup analysis, we adjusted all the confounders of model 4 except for the stratified factors.

**Table 4:** Incremental predictive value of CVAI in risk assessment of CAS among steelworkers.

Model	C-Statistic (95%CI)	*p*-Value	IDI (95%CI)	*p*-Value	NRI (95%CI)	*p*-Value
Basic model	0.775 (0.759–0.791)	Ref.	Ref.		Ref.	
+CVAI	0.804 (0.789–0.819)	<0.001	0.0313 (0.0251–0.0375)	<0.001	0.0714 (0.0366–0.1062)	<0.001

Basic model included age, sex, family history of CAS, smoking, drinking, physical exercise, diet, hypertension, diabetes, dyslipidemia, uric acid, homocysteine, BMI, exposure to high temperature, noise, and shifts. CI: confidence interval, CVAI: Chinese Visceral Adiposity Index, IDI: integrated discrimination improvement, NRI: net reclassification improvement.

## Data Availability

The datasets generated and analyzed during the current study are available from the corresponding author upon reasonable request.
